# Liquid-Based Cytopathology Test: A Novel Method for Diagnosing Pulmonary Mucormycosis in Bronchial Brushing Samples

**DOI:** 10.3389/fmicb.2018.02923

**Published:** 2018-11-27

**Authors:** Xiaolin Jiang, Tian Yang, Qiyuan Li, Xianglan Zhu, Xueying Su, Jinnan Li, Yong Jiang

**Affiliations:** Department of Pathology, West China Hospital of Sichuan University, Chengdu, China

**Keywords:** liquid-based cytopathology test, conventional cytopathology, mucormycosis, Mucorales, bronchial brushing sample

## Abstract

**Background:** Pulmonary mucormycosis, a relatively rare but severe pulmonary fungal disease with a high mortality rate, is difficult to diagnose in immunocompromised patients. Conventional cytopathology (CCP) examination of respiratory samples can help detect Mucorales, but its diagnostic sensitivity is poor. The aim of this study was to assess the first application of liquid-based cytopathology test (LCT) to detect Mucorales.

**Methods:** A total of 33 pairs of bronchial brushing samples from 27 patients diagnosed as pulmonary mucormycosis by fiberoptic bronchoscopy biopsy were prepared as slides using both CCP and LCT. LCT and CCP used the same cytology brush to obtain samples at the same site during the same time as the fiberoptic bronchoscopy biopsy. All samples were stained with Papanicolaou, GMS and PAS. CCP and LCT slides were evaluated from the rate of positive detection, 8 cytomorphological features and 7 background features.

**Results:** LCT-prepared slides showed a higher positive rate of Mucorales detection than CCP-prepared slides for Papanicolaou’s staining [28/33 (84.85%) vs. 15/33 (45.45%), *p* = 0.001] and for “special staining” with GMS and PAS [29/33 (87.88%) vs. 18/33 (54.55%), *p* = 0.003]. Clearer smear background and more distinct stereoscopic cytopathological features were observed in LCT. Messy yarn-like necrosis observed in conventionally prepared 75.76% (25/33) samples was cytomorphological suggestive for the diagnosis of mucormycosis.

**Conclusion:** This retrospective study suggests that LCT may be better than CCP to detect Mucorales in bronchial brushing samples from patients with pulmonary mucormycosis.

## Introduction

Mucorales, a widespread filamentous order of fungi in the environment, can cause rare but severe infection in immunocompromised patients with hematological malignant disease, uncontrolled diabetes mellitus, trauma, organ transplants, allogeneic stem cell transplantation, renal disease, septicemia, burns, malnutrition, etc. (Roden et al., 2005; Nasa et al., 2017). Mucormycosis, a life-threatening opportunistic fungal infection, is classified into six forms: rhinocerebral, pulmonary, cutaneous, gastrointestinal, disseminated, and uncommon presentations (Petrikkos et al., 2012). Pulmonary mucormycosis is the second most common presentation (Lin et al., 2017), representing up to 80% of the mortality rate due to delay diagnosis and inadequate treatment (Tedder et al., 1994).

So far, laboratory methods for diagnosing mucormycosis include conventional fungal culture, histopathologic examination, cytology, serology, and molecular-based diagnosis. Most diagnostic approaches are still in their infancy, suffering either from long-lasting protocol, poor specificity and/or sensitivity, leading to erroneous or delayed diagnosis (Lackner et al., 2014). Direct microscopy, histopathology and culture are strongly recommended by the European Society of Clinical Microbiology and Infectious Diseases and the European Confederation of Medical Mycology Joint Clinical Guidelines (Cornely et al., 2014). Nevertheless, there are challenges to establish a clinical diagnosis of mucormycosis due to the difficulty in obtaining a clear proof of culture in some cases and the fact that histology is an invasive procedure not suitable for some cases.

Cytopathology, a branch of clinical pathology, is similar to direct microscopy in operational procedures and techniques, having received increasing attention in the examination of fungal diseases because of its rapidity, accuracy, and minimal invasiveness. It is located at the same position as histopathology and fungal culture in the Practice Guidelines for the Diagnosis and Management of Aspergillosis (Patterson et al., 2016). Cytopathological detection of Mucorales in respiratory tract samples plays a major role in accurate diagnosis based on subsequent culture or histology examination. In conventional cytopathological (CCP) test, fresh tissue sample is placed on a glass slide, immersed in wet medium, stained by Papanicolaou or “special staining,” usually Gomori’s methenamine silver (GMS) or periodic acid-Schiff (PAS), and finally the slide is covered. However, it has limitations that there are stacks of inflammatory cells, hemorrhagic background, and necrotic tissue, as well as air-drying artifacts, protein/mucus and oversized smears, resulting in poor sensitivity of the CCP (Celik et al., 2008).

Liquid-based cytopathology test (LCT), developed in 1991, improves the quality of samples and effectiveness of cytopathological tests. Since then, it has been widely utilized in various cancers, including breast, lung as well as cervical cytology (Hoda et al., 2013; Gerhard and Schmitt, 2014; Michael and Bedrossian, 2014). With the advantages of standardized and automated preparation, it has reduced unsatisfactory rate and improved specimen adequacy and the ability to perform ancillary test with residual specimen (Fremont-Smith et al., 2004). So, it is a more sensitive, specific and cost-effective counterpart than conventional cytopathology (Cox, 2004). Recently, LCT has already been developed and applied to the diagnosis of pulmonary aspergillosis (Shen et al., 2017). LCT may be extremely useful for the rapid diagnosis of pulmonary mucormycosis as these organisms are fatal to the patient’s survival. This study was the first attempt to evaluate the applicability of LCT in bronchial brushing samples to detect Mucorales by Papanicolaou’s staining and “special staining” with GMS and PAS vs. conventional cytology.

## Materials and Methods

### Patient Selection

Ethical approval was obtained from the West China Hospital Ethics Committee of Sichuan University and was conducted in accordance with the latest version of the Helsinki Declaration. According to local ethics, we have applied for exemption from written informed consent.

In this retrospective study, 32 patients, whose fiberoptic bronchoscopy biopsy samples were diagnosed pulmonary mucormycosis by histopathology, were retrieved in medical record system of West China Hospital between January 2013 and July 2018. Patients who did not have bronchial brushing samples (including CCP and LCT) at the same site during the same time as the fiberoptic bronchoscopy biopsy were excluded. Finally, 27 diagnosed cases of pulmonary mucormycosis were included in the study. All histopathological slides from each patient were reviewed and confirmed mucormycosis according to ESCMID and ECMM joint clinical guidelines for the diagnosis and management of mucormycosis 2013 (Cornely et al., 2014) by two professional and experienced pathologists with consistent diagnosis independently and in duplicate. The histopathological features that allow diagnosis include; non-septate or pauci-septate, irregular, ribbon-like hyphae, angle of branching 45–90°, hyphal diameter 6 to >16 μm.

A total of 33 pairs of bronchial brushing samples (4 patients had 2 distinct pairs and 1 patient had 3 distinct pairs, each with corresponding bronchial brushing samples) for CCP and SurePath LCT were available and finally included in this study.

### Papanicolaou’s Staining

LCT and CCP used the same method from the same cytology brush to obtain samples.

For conventional processing, after fiberoptic bronchoscopy biopsy, two direct smears were prepared using disposable cytology brush [Micro-Tech (Nanjing), China] and fixed immediately in 95% ethanol and stained with Papanicolaou (Pap) techniques.

For LCT, after two direct smears were prepared using disposable cytology brush, residues of the cytology brush were washed and transferred to a small bottle with 10 mL of CytoRich^TM^ medium and incubated at room temperature for 30 min, followed by centrifugation at 600 rpm for 5 min. The supernatant was removed and the pellet was vortexed and transferred to the AutoCyte PREP system (TriPath Imaging), in which slides were automatically prepared and stained with Papanicolaou. LCT processing was carried out by using SurePath LCT Kit (TriPath PREP, BD SurePath, Burlington, NC, United States).

### “Special Staining” With GMS and PAS

After Papanicolaou’s staining, all samples were stained with GMS and PAS. CCP samples with Papanicolaou’s staining required fading treatment and then stained with GMS and PAS. LCT samples from residues of liquid-based bottles were prepared directly using GMS and PAS. Special Stains Automated Slide Stainer (NEXES, United States) was used with a commercial kit (Roche Diagnostics, United States).

### Cytopathological Examination

All cytologic slides were screened and assessed independently by two cytologists with more than 5 years of experience. Slides stained with Papanicolaou were considered as mucormycosis if hyphae fungi are typically broad, ribbonlike, and irregularly shaped, non-septate or sparsely septate, with “right angles” branches. Slides stained with PAS or GMS were considered, respectively, positive if magenta or brown-black fungal hyphae with morphological features as mentioned before were observed. But the Mucorales was detected in “special staining” only if both GMS- and PAS-staining were positive. Disagreements were settled through discussion. Both conventional and LCT slides stained with Papanicolaou’s staining were systematically evaluated for 8 cytomorphologic features which were related to the diagnosis of Mucorales including staining quality (not good, staining in <50% mycelium; good, staining in ≥50% mycelium), right angle branches (not obvious, in <50% mycelium; obvious, in ≥50% mycelium), mycelium wall (not clear, visible in <50% mycelium; clear, visible in ≥50% mycelium), wrinkle (not obvious, in <50% mycelium; obvious, in ≥50% mycelium), septum (non-septate or sparsely septate), number of mycelium (obviously increase, ≥50% mycelium relative to paired CCP samples), and presence or absence of messy yarn-like necrosis and transitional Mucorales. They were also studied 7 background features including clean, inflammation, necrosis, protein/mucus, thick smear, hemorrhage, and air dried/poor fixation (Celik et al., 2008; Hoda et al., 2013).

### Statistical Analysis

The sensitivity of the two methods was compared with the *χ*^2^-test using the SPSS 18.0 for Windows (IBM, Chicago, IL, United States). Categorical data were expressed as absolute or relative frequencies, and continuous data were expressed as mean ± SD. Statistical significance was defined as a two-sided *P* < 0.05.

## Results

The study involved 27 patients (F/M, 4/23; age, 55.1 ± 12.1 years) with following comorbidities: 18 (66.67%) had diabetes, 6 (22.22%) had solid tumor (lung cancer), 2 (7.41%) had hematological malignancy, 1 (3.70%) had renal transplant, and 8 (29.63%) had others. The location of fiberoptic bronchoscopy biopsy was 19 cases of right lung and 8 cases of left lung. Positive culture cases were 4/22. Positive 1, 3-beta-D-glucan assay cases were 3/19. Positive Galactomannan assay cases were 8/19. All patients were negative for sputum smear. CT of the chest was obtained in 96.30% of patients. There was a wide spectrum of radiological findings (Table 1), with the most common being cavitation, followed by nodules, mass, consolidation, and pleural effusion. Based on the above clinical examination, the clinician has a preliminary suspicious’ diagnosis about pulmonary disease before the fiberoptic bronchoscopy: 14 were pulmonary infection which may be bacterial, tuberculosis, and do not exclude fungal infections, 5 were mycosis, 4 were lung cancer, 3 were tuberculosis, 1 was pulmonary abscess. Result of bronchofibroscopy were obtained in all patients: 8 patients were normal, and 22 patients were mainly necrosis and Luminal stenosis. At the time of bronchofibroscopy, 6 patients were submitted lavage specimens. Only one was diagnostic and the rest five was negative.

**Table 1:** Clinical and biological data from patients with pulmonary mucormycosis.

Patient	Underlying	Location of	Sputum	1, 3-beta-	Galacto	CT finding	Pulmonary	Broncho	Cyto	Lavage
No.	disease	Pulmonary	culture	D-glucan	mannan		disease^a^	fibroscopy	frequency	Sample
		infection		assay	assay					
1	LC	SR	ND	ND	ND	ND	TB	Luminal stenosis	1	NO
2	LC	IR	ND	ND	ND	Mass	LC	Normal	1	NO
3	Bronchiectasis, diabetes	IR	Negative	Negative	ND	Cavitation	Mycosis	Luminal stenosis	2	NO
4	LC	SL	Negative	ND	ND	Consolidation	Pulmonary infection^b^	Purulent secretion	1	NO
5	CKD, diabetes	SR	Negative	Negative	ND	Cavitation	Pulmonary infection	Luminal stenosis	1	NO
6	Diabetes	SR	*Rhizomucor* sp	Negative	Negative	Cavitation	Pulmonary infection	Normal	1	Negative
7	Diabetes	IL	Negative	Negative	Negative	Mass	Pulmonary abscess	Necrosis	1	NO
8	CKD, diabetes	SR	Negative	Negative	Negative	Nodule, pleural effusion	Pulmonary infection	Luminal stenosis	1	NO
9	Diabetes	SR	Negative	Negative	Negative	Nodule, Cavitation	TB	Necrosis	1	NO
10	LC	MR	Negative	Negative	Negative	Cavitation	Mycosis	Normal	1	NO
11	Diabetes	IR	Negative	Negative	Negative	Consolidation	TB	Necrosis	1	NO
12	Diabetes	IL	Negative	Negative	Negative	Nodule	Mycosis	Necrosis	1	NO
13	Diabetes	SL	Negative	Negative	Negative	Mass, Cavitation	Pulmonary infection	Necrosis	1	NO
14	COPD	IR	Negative	Negative	Negative	Mass	Pulmonary infection	Luminal stenosis	2	Negative
15	COPD	SR	*Rhizopus* sp	Positive	Positive	Cavitation	Mycosis	Normal	3	Negative
16	Diabetes	SR	Negative	Negative	Negative	Cavitation	Pulmonary infection	Necrosis	1	Negative
17	LC	SL	ND	ND	ND	Nodule	LC	Normal	1	NO
18	CKD, diabetes	SR	Negative	ND	Positive	Cavitation	Pulmonary infection	Luminal stenosis	1	NO
19	COPD, Diabetes	IR	Negative	Negative	Positive	Mass	Pulmonary infection	Necrosis	1	NO
20	ALL, diabetes	SR	ND	ND	ND	Mass	Pulmonary infection	hemorrhage	1	NO
	Renal transplant									
21	LC	IR	ND	ND	ND	Nodule	LC	Purulent secretion	1	NO
22	CKD, diabetes	SL	Negative	Negative	Negative	Nodule, Cavitation	LC	Normal	1	NO
23	Diabetes	IL	*Lichtheimia corymbifera*	Positive	Positive	Nodule	Pulmonary infection	Purulent secretion	1	NO
24	Diabetes	IL	*Lichtheimia corymbifera*	Positive	Positive	Nodule	Pulmonary infection	Neoplasm	2	Negative
25	Diabetes	MR	Negative	Negative	Positive	Cavitation	Pulmonary infection	Normal	1	NO
26	Diabetes	SR	Negative	ND	Positive	Nodule, Cavitation	Mycosis	Necrosis	2	NO
27	AML	IR	Negative	Negative	Positive	Consolidation	Pulmonary infection	Normal	1	Mucormycosis

*LC, lung cancer; CKD, chronic kidney disease; COPD, chronic obstructive pulmonary disease; ALL, acute lymphoblastic leukemia; AML, acute myeloblastic leukemia; SL, superior lobe of left lung; IL, inferior lobe of left lung; SR, superior lobe of right lung; MR, middle lobe of right lung; IR, inferior lobe of right lung; ND, not done; NA, not available; TB, tuberculosis; ^*a*^pulmonary disease suspected by the clinician before fiberoptic bronchoscopy; ^*b*^pulmonary infection, not exclude fungal infections*.

In Papanicolaou’s staining, the LCT platform detected Mucorales in 28 of 33 samples, corresponding to 84.85% sensitivity. This was significantly higher than CCP (15 of 33, 45.45%, *P* < 0.005). In “special staining,” the rate of positive detection between LCT and CCP samples was different [87.88% (29/33) vs. 54.55% (18/33)], which was a statistical significance (*P* < 0.005). There were 3 samples in the CCP, and 1 of the LCT was not diagnosed by Papanicolaou’s staining, but diagnosed by “special staining.” *P*-values were 0.460 and 0.720, which were not statistically significant (Table 2).

**Table 2:** Results of conventional and liquid-based cytopathology.

Staining	Positive cases-CCP	Positive cases-LCT	*P*-value
	(*n* = 33)	(*n* = 33)	
Papanicolaou’s	15 (45.45%)	28 (84.85%)	0.001
“Special staining”	18 (54.55%)	29 (87.88%)	0.003
*P*-value	0.460	0.720	

*LCT, liquid-based cytopathology; CCP, conventional cytopathology*.

**FIGURE 1:**
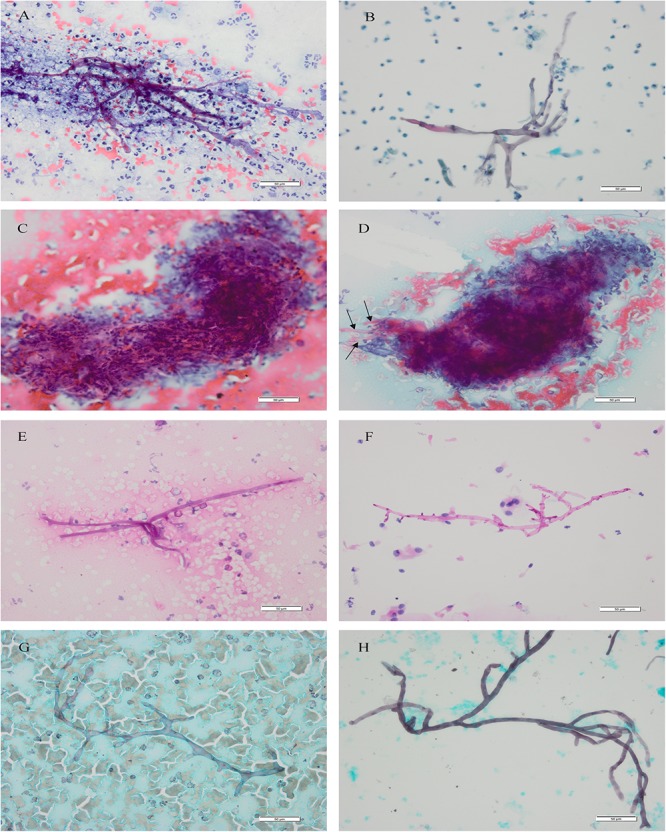
Mucorales. Cytomorphology of Mucorales in bronchial brushing samples processed using the SurePath LCT platform or CCP, followed by Papanicolaou’s staining, PAS staining and GMS staining. Cytopathological features of Mucorales filaments included typical diameter, ribbonlike, non-septate or sparsely septate, and dichotomous branching at right angles. Magnification, 400×. **(A)** CCP by Papanicolaou’s staining; **(B)** LCT by Papanicolaou’s staining; **(C)** messy yarn-like necrosis: cytomorphology of “messy yarn-like necrosis” in bronchial brushing samples processed using the CCP, followed by Papanicolaou’s staining; **(D)** transitional Mucorales (arrows): Mucorales which were transitional with the outlines of messy spin can be seen around the “messy yarn-like necrosis”; **(E)** CCP by PAS staining; **(F)** LCT by PAS staining; **(G)** CCP by GMS staining; **(H)** LCT by GMS staining. The mycelium of Mucorales appeared magenta after PAS staining and brown-black after GMS staining. CCP, conventional cytopathology; LCT, liquid-based cytopathology test; PAS, Periodic Acid-Schiff; GMS, Gomori’s Methenamine Silver. Scale bar: 50 um.

In Papanicolaou’s staining, the cytomorphologic features about Mucorales in LCT samples (*n* = 28) were clearer and easier to detect than in CCP samples (*n* = 15). Pale blue or pink Mucorales filaments showed typical diameter (4∼25 um), ribbonlike, non-septate or sparsely septate, and dichotomous branching at right angles (Figures 1A,B). The staining quality was “good” in 66.67% of CCP and 96.43% of LCT with a *P*-value = 0.026; the right-angle branches were “obvious” in 53.33% of CCP and 92.86% of LCT with a *P*-value = 0.008. They were statistically significant, which showed better staining of the mycelium and been clearer to see the branches in LCT. In CCP, the mycelium wall was “clear” in 66.67% of the samples, the wrinkle was “obvious” in 60.00%, and the septum was “sparsely septate” in 20.00%. Whereas, in LCT, the mycelium wall was “clear” in 89.29% of the samples, the wrinkle was “obvious” in 85.71%, and the septum was “sparsely septate” in 46.43%. Their *P*-values were >0.05 implying a lack of statistically significant difference between the two methods, with regard to mycelium wall, wrinkle, and septum (Table 3). In six LCT samples the number of mycelia obviously increased relative to paired CCP samples.

**Table 3:** Cytomorphologic features about Mucorales in Papanicolaou’s staining.

Cytomorphologic	Variables	CCP	LCT	*P*-value
features		(*n* = 15)	(*n* = 28)	
Staining quality	Good	10 (66.67%)	27 (96.43%)	0.026
	Not good	5 (33.33%)	1 (3.57%)	
Right angle branches	Obvious	8 (53.33%)	26 (92.86%)	0.008
	Not obvious	7 (46.67%)	2 (7.14%)	
Mycelium wall	Clear	10 (66.67%)	25 (89.29%)	0.160
	Not clear	5 (33.33%)	3 (10.71%)	
Wrinkle	Obvious	9 (60.00%)	24 (85.71%)	0.128
	Not obvious	6 (40.00%)	4 (14.29%)	
Septum	Sparsely septate	3 (20.00%)	13 (46.43%)	0.087
	Non-septate	12 (80.00%)	15 (53.57%)	

*LCT, liquid-based cytopathology; CCP, conventional cytopathology*.

A proportion of Papanicolaou’s staining samples showed distinctive necrosis that we termed “messy yarn-like necrosis,” which appeared under the microscope as spinning necrosis that gathered into a rounded mass. Its messy spinning outlines were continuously visible in the necrosis (Figure 1C). This necrosis was only observed in conventionally prepared 75.76% (25/33) samples. Around the “messy yarn-like necrosis,” we observed Mucorales which were transitional with the outlines of messy spin (Figure 1D) in the 52.00% (13/25) cases.

The obscuring factors were studied in all the samples with Papanicolaou’s staining by observing the overlapping cluster of inflammatory cells, necrosis, blood cell and protein/mucus in background, thick smear, and air dried/poor fixation (Rosa et al., 2013). The characteristic background features observed in samples prepared by the methods of CCP and LCT were summarized in Table 4. In CCP, the clean was in 9.09%, inflammation in 84.85%, necrosis in 87.88%, protein/mucus in 27.27%, thick smear in 30.30%, hemorrhagic background in 42.42%, and air dried/poor fixation in 9.09%. Whereas, in LCT, the background was clean in 72.73%, inflammation in 24.24%, necrosis in 21.21%, protein/mucus in 3.03%, and thick smear in 6.06%. Inflammatory cells, necrosis, protein/mucus, and thick smear were significantly reduced and evenly distributed. Their *P*-values were <0.05 which was statistically significant. None had a hemorrhagic background and air dried/poor fixation. The microscope fields in LCT samples were generally clearer than conventional slides, showing less necrosis, mucus, inflammatory cells and blood. Similar results of LCT-prepared slides were observed after Specific staining (Figures 1E–H).

**Table 4:** Background studies in Papanicolaou’s staining.

Background	CCP	LCT	*P*-value
	(*n* = 33)	(*n* = 33)	
Clean	3 (9.09%)	24 (72.73%)	<0.001
Inflammation	28 (84.85%)	8 (24.24%)	<0.001
Necrosis	29 (87.88%)	7 (21.21%)	<0.001
Protein/mucus	9 (27.27%)	1 (3.03%)	0.006
Thick smear	10 (30.30%)	2 (6.06%)	0.011
Hemorrhage	14 (42.42%)	0	–
Air dried/poor fixation	3 (9.09%)	0	–

*LCT, liquid-based cytopathology; CCP, conventional cytopathology*.

## Discussion

To identify Mucorales, direct culturing, which is time-consuming and often presents negative results, is not suitable for rapid diagnosis of mucormycosis on respiratory tract samples, and only 4 of them are positive in this study. Moreover, Serology-based diagnostic assays such as the serum galactomannan and β-glucan assay are highly suggestive of fungal pneumonia other than mucormycosis (Hamilos et al., 2011). Unfortunately, detection of specific antigens or nucleic acid by polymerase chain reaction (PCR) are currently rarely used to diagnose mucormycosis and are still limited in experiments. High numbers of patients with positive galactomannan assay were 8/19, 6 pulmonary infections and 2 mycoses, which are inconsistent with the diagnosis of mucormycosis. Their pulmonary diseases were suspected by the clinician before fiberoptic bronchoscopy. As reported in the literature, cross-reaction from an existing non-Aspergillus fungal infection may result in false-positive GM (Martin-Rabadan et al., 2012). False positive reactions also may be due to the presence of GM in blood-derived products, gluconate sodium-containing hydration solutions, antibiotics or food products (Ansorg et al., 1997; Petraitiene et al., 2011; Martin-Rabadan et al., 2012). Histological examination of biopsied tissue are the preferred diagnostic methods, but are variably invasive and available only at special institutions. For example, diabetics, the most prevalent underlying condition, have an apparent predilection for developing endobronchial lesions (including mucormycosis), which can invade major pulmonary blood vessels; patients usually die from massive hemoptysis (Donohue, 1983). Although direct microscopy can be performed quickly, hyphae are easily obscured by inflammatory cells and necrosis in the background, resulting in a low positive rate as well as conventional cytology smears. These deficiencies of all the above methods have led to an effort to develop new methods for rapid identification of Mucorales.

Patients with pulmonary mucormycosis require early and accurate diagnosis in order to receive their timely and optimal anti-fungal treatment (De Pauw et al., 2008). If treatment is not initiated promptly, Mucorales species may cause acute and highly invasive disease in predisposed patients (Prabhu and Patel, 2004). Stained cytological slides play an important role, including conventional and liquid-based cytological preparation, but conventional preparation is still a great challenge to detect Mucorales due to its several disadvantages as shown in other literatures or specimens (Ribes et al., 2000; Sangoi et al., 2009; Tathe et al., 2016). In this study, we compared the ability of LCT and CCP to allow Mucorales detection in bronchial brush samples after Papanicolaou’s staining or “special staining.” The results showed that LCT had a higher positive rate of Mucorales detection than CCP samples. Similar results demonstrated that LCT-prepared slides showed a higher positive rate of Aspergillus detection in bronchial brushing samples (83.33 vs. 57.41%, *P* < 0.05) (Shen et al., 2017). The LCT also had a significantly higher diagnostic sensitivity for lung cancer (80.2%) than the conventional PS (pick-and-smear) method (63.4%, *P* < 0.05) (Wu et al., 2009). We believe that LCT can accurately and promptly diagnose pulmonary mucormycosis. However, in LCT, there are still 5 Papanicolaou’s staining and 4 “special staining” samples that do not detect Mucorales. We reviewed the histology biopsy from the same site and found that the number of Mucorales was initially small.

This is the first attempt to compare the morphology of Mucorales in LCT and CCP. The LCT platform offers advantages over CCP about cytomorphology. Our study shows that LCT improves the visualization of Mucorales. There is a statistically significant difference between the two methods regarding the quality of staining which is better in LCT. When quality of Papanicolaou’s staining is “not good,” the amount of Mucorales that can be observed is reduced. So, “special staining” will play a certain role in increasing the detection of Mucorales. The right-angle branches of Mucorales also proved to be a significant morphological feature. Branches of Mucorales, often irregular but mainly right-angled, in particular assume great importance in the morphology diagnosis of Mucormycosis. There is no statistically significant difference between the two methods regarding the mycelium wall, wrinkle, and septum. However, identification of these features relies on well-fixed, well-prepared, and well-stained smears. In fact, these features, when be observed, can easily tilt the diagnosis toward Mucorales.

CCP samples revealed a potentially novel type of necrosis with definite cytomorphologic features that differ from usual caseous necrosis (complete and granular) and tumor necrosis (characteristic pyknotic tumor cell nucleus). In 75.76% (25/33) samples, we observed that the necrosis presented disorderly spinning, which we named “messy yarn-like necrosis.” Since the pathological nature of mucormycosis is purulent inflammation and hemorrhagic necrotizing inflammation, there are excessive necrosis and inflammatory cells in cytopathological specimens. Moreover, in conventionally prepared paired samples with such necrosis, Mucorales was discovered after LCT which had removed necrotic substances. More importantly, we observed in the 13/25 cases that the spinning outlines of the necrosis and the Mucorales hyphae were transitional. We suggest that hyphae of Mucorales, which do not have a definite direction in itself, are masked by this necrosis, so the details of the hyphae are not clearly observed. Therefore, “messy yarn-like necrosis” on conventional samples is suggestive for the diagnosis of Mucormycosis.

Regarding the background of the slides, 72.73% of LCT showed a clean background in sharp contrast to 9.09% of CCP, with a *P*-value of <0.001. Similar findings were reported in most other studies (Hong et al., 2014; Dadhich et al., 2016). In CCP, the unclean background is caused by various obstructive factors, which have been largely eliminated by LCT. In LCT, cytopathology slides have uniform thickness; cellular structure is well preserved; poor fixation and hemorrhage are absent; inflammatory cells, necrosis, and protein/mucus are significantly reduced; screening area is made smaller and hence screening time is reduced. We found 9 LCT cases with obstructive factors in the background slide, including 4 cases of inflammatory cells and necrosis; 2 of inflammatory cells; 1 of necrosis; 1 of inflammatory cells, necrosis and thick smear; 1 of inflammatory cells, necrosis, thick smear, and protein/mucus. We presumed that inflammatory cells and necrosis may be the major obscuring factors in LCT. Therefore, background factors affect not only the diagnosis of Mucorales, but also the clear identification of the morphological characteristics of Mucorales as described above.

Our results suggest that LCT is a novel method to detect Mucorales in the respiratory tract. Residues from liquid-based bottles can be used for subsequent auxiliary tests, including “special staining,” immunolabeling, and molecular testing, while there are many restrictions on obtaining materials from CCP slides for subsequent testing. In addition, automatic preparation of LCT slides and the staining of the AutoCyte PREP system help to ensure consistent quality of results, regardless of practitioners’ experience. However, some limitations and several considerations indicate that it has some drawbacks in the diagnosis of pulmonary mucormycosis alone. First, LCT cannot accurately identify the type of Mucorales species, which may affect the selection of antifungal drug. Identifying Mucorales organisms to genus or species levels brings valuable epidemiological, therapeutic and prognostic implications (Gomes et al., 2011). Liposomal amphotericin B is strongly recommended as a first-line antifungal treatment (Cornely et al., 2014). Therefore, species identification has little effect on the initial treatment. The inability to identify Mucorales species is not only an inherent defect in cytopathology or even histopathology. It is expected that species will be identified by subsequent PCR assays based on cytological samples (Dannaoui et al., 2010). Second, LCT may produce false-negative results when a small amount of fungi is present or the site of infection is not connected to the bronchus (Shen et al., 2017). Third, the positive detection rate of Papanicolaou’s staining and “special staining” showed no significant difference. However, the British Society for Medical Mycology best practice recommends “special staining” should be performed on all available samples and even used routinely in samples from immunocompromised patients in order to maximize the possibility of identifying Mucorales (Schelenz et al., 2015).

In conclusion, this preliminary study shows that LCT-based Papanicolaou’s staining and specific staining may be helpful in detecting Mucorales with higher sensitivity, better cytomorphologic features, and clearer smear background in bronchial brushing samples from patients with pulmonary mucormycosis. In the future, liquid-based assays may be extended to other respiratory cytology samples of patients suspected of having mucormycosis, especially those who cannot tolerate or obtain biopsy, such as sputum, bronchial washing, and bronchoalveolar lavage fluid. Since LCT is similar to direct microscopy, LCT can also be used as a reference for the diagnosis of fungi in clinical microbiology laboratories to improve sensitivity.

The results of our samples should be validated in multicenter studies because of the rare occurrence of mucormycosis, to develop tests for this clinical application. The results of retrospective studies described here are based on histopathological diagnosed patients with pulmonary mucormycosis, enabling us to calculate only positive detection (sensitivities) as a partial measure of diagnostic performance.

## Author Contributions

YJ and XJ conceived and designed the experiments and wrote the manuscript. TY, QL, XZ, XS, and JL analyzed the data and reviewed the manuscript.

## Conflict of Interest Statement

The authors declare that the research was conducted in the absence of any commercial or financial relationships that could be construed as a potential conflict of interest.
